# Improving Coccidioidomycosis Testing for Emergency Department Patients With Suspect Community-Acquired Pneumonia: Analysis of Provider Attitudes and the Effect of a Targeted Intervention

**DOI:** 10.1093/ofid/ofae461

**Published:** 2024-08-12

**Authors:** Cody A Cunningham, Ashlyn T Brown, Srekar N Ravi, Jeremiah J Bearss, Michael P O’Shea, Amani K Elshaer, Matt V Biondi, Bobak Seddighzadeh, Sandra N Elmasry, Amogh Havanur, Avanika Mahajan, Juliana Savic, Nneoma U Alozie, Douglas Rappaport, Andrej Urumov, Janis E Blair

**Affiliations:** Department of Internal Medicine, Mayo Clinic School of Graduate Medical Education, Scottsdale, Arizona, USA; Department of Internal Medicine, Mayo Clinic School of Graduate Medical Education, Scottsdale, Arizona, USA; Department of Internal Medicine, Mayo Clinic School of Graduate Medical Education, Scottsdale, Arizona, USA; Department of Internal Medicine, Mayo Clinic School of Graduate Medical Education, Scottsdale, Arizona, USA; Department of Internal Medicine, Mayo Clinic School of Graduate Medical Education, Scottsdale, Arizona, USA; Department of Internal Medicine, Mayo Clinic School of Graduate Medical Education, Scottsdale, Arizona, USA; Department of Internal Medicine, Mayo Clinic School of Graduate Medical Education, Scottsdale, Arizona, USA; Department of Internal Medicine, Mayo Clinic School of Graduate Medical Education, Scottsdale, Arizona, USA; Department of Internal Medicine, Mayo Clinic School of Graduate Medical Education, Scottsdale, Arizona, USA; Department of Internal Medicine, Mayo Clinic School of Graduate Medical Education, Scottsdale, Arizona, USA; Department of Internal Medicine, Mayo Clinic School of Graduate Medical Education, Scottsdale, Arizona, USA; Department of Internal Medicine, Mayo Clinic School of Graduate Medical Education, Scottsdale, Arizona, USA; Department of Internal Medicine, Mayo Clinic School of Graduate Medical Education, Scottsdale, Arizona, USA; Department of Emergency Medicine, Mayo Clinic Arizona, Phoenix, Arizona, USA; Department of Emergency Medicine, Mayo Clinic Arizona, Phoenix, Arizona, USA; Division of Infectious Disease, Mayo Clinic Arizona, Phoenix, Arizona, USA

**Keywords:** coccidioidal serologic testing, coccidioidomycosis, community acquired pneumonia, emergency department

## Abstract

Coccidioidomycosis is a common cause of community-acquired pneumonia in endemic regions. Approximately 20 000 cases of coccidioidomycosis occur annually; however, this statistic is limited by a widespread lack of testing. Here, we analyze emergency medicine provider attitudes toward coccidioidal testing and assess the effect of an intervention to improve testing rates.

Coccidioidomycosis is a febrile respiratory infection caused by the dimorphic fungi *Coccidioides immitis* and *Coccidioides posadasii* [1]. While generally asymptomatic or self-limited, coccidioidomycosis can progress to disseminated disease, especially in patients with active immunosuppression, pregnancy, or certain inborn errors of immunity, and it is associated with significant morbidity and mortality rates [2, 3]. This disease is highly endemic to the southwestern United States, especially in Arizona and California, where an estimated 17.3%–26.0% of community-acquired pneumonia (CAP) cases are attributable to *Coccidioides* [4], accounting for about 22 061 cases annually [5]. However, these studies likely underestimate the total number of coccidioidal cases annually due to false-negative test results early in the disease course [6, 7] and a widespread lack of coccidioidal serologic testing (CST) in highly endemic regions [8]. One group found that 2%–13% of ambulatory patients with CAP were tested for coccidioidomycosis [9]. A more recent study focused on urgent cares in a highly endemic region found testing rates to be 7.2%–22% in patients with suspected pneumonia [4].

Failure to test for coccidioidomycosis results in patients experiencing a protracted illness course before receiving the appropriate diagnosis, and patients were symptomatic for a mean (interquartile range) of 23 (6–74) days before receiving the diagnosis of coccidioidomycosis [10]. Incorrect diagnoses lead to repeated presentations to medical care and courses of unneeded antibiotics. They also puts high-risk patients at risk for disseminated disease [3].

An increasing percentage of patients seek care in the emergency department (ED), accounting for a total of 139.8 million visits annually [11]. Indeed, 23% of patients ultimately receiving a diagnosis of coccidioidomycosis first presented to medical attention in an ED, and 46% of patients reported ≥1 ED visit during the course of illness [10]. We devised the current study to evaluate ED physician attitudes toward CST and to evaluate the effect of a targeted educational intervention on the rates of CST in our tertiary medical center ED.

## METHODS

### ED Physicians Survey

A 12-item electronic survey assessing attitudes toward CST was sent to the 37 ED physicians at our tertiary medical center, using Microsoft Forms. For multiple-choice question, multiple selections could be made or left blank. Results were exported to Microsoft Excel for analysis.

### Intervention

The targeted intervention took place on 1 November 2023 during a regularly scheduled emergency medicine department meeting, with a hybrid of in-person and virtual attendance. Three authors (C. A. C., J. J. B., and J. E. B.) led a discussion surrounding the epidemiology, diagnosis, and management of coccidioidomycosis. Additional stakeholder feedback was solicited. To reenforce the concepts presented in the educational intervention, ED physicians were introduced to the Centers for Disease Control and prevention (CDC)–sponsored “Community-Acquired Pneumonia: When To Think Fungus” module (http://funguscme.org/CAP2022/). As an incentive to complete the module, physicians were offered nominal compensation in the form of a Starbucks gift card.

### Data Acquisition

Two tools were developed to track CST order counts over time using a standard query language report from the Epic electronic medical record (EMR) used at our institution. The percentage of CAP with CST orders was calculated by tallying the ED outpatients who were assigned a pneumonia code from the *International Classification of Diseases, Ninth Revision* (J18.0–J18.9 and J15.0–J15.9), with a secondary query tallying whether or not *Coccidioides* immunoglobulin (Ig) M/IgG by enzyme immunoassay with reflex to complement fixation and immunodiffusion (termed CST; Current Procedural Terminology, code 86635) was ordered for the episode of care. The count of all CST orders in the ED for any indication (ie, not limited to a CAP diagnosis) was normalized by the number of patient visits to the ED over the same time period. “Preintervention” was defined as 1 August 2022 to 31 October 2023; “postintervention,” as 1 November 2023 to 30 April 2024.

### Consent and Ethical Considerations

The Mayo Clinic Institutional Review Board (IRB) reviewed ethical considerations for this project, which, as a quality improvement project designed to improve the rate of CST orders, was deemed not research and requiring no further IRB considerations (IRB no. 23-001817; 13 March 2023).

## RESULTS

**Figure 1.**
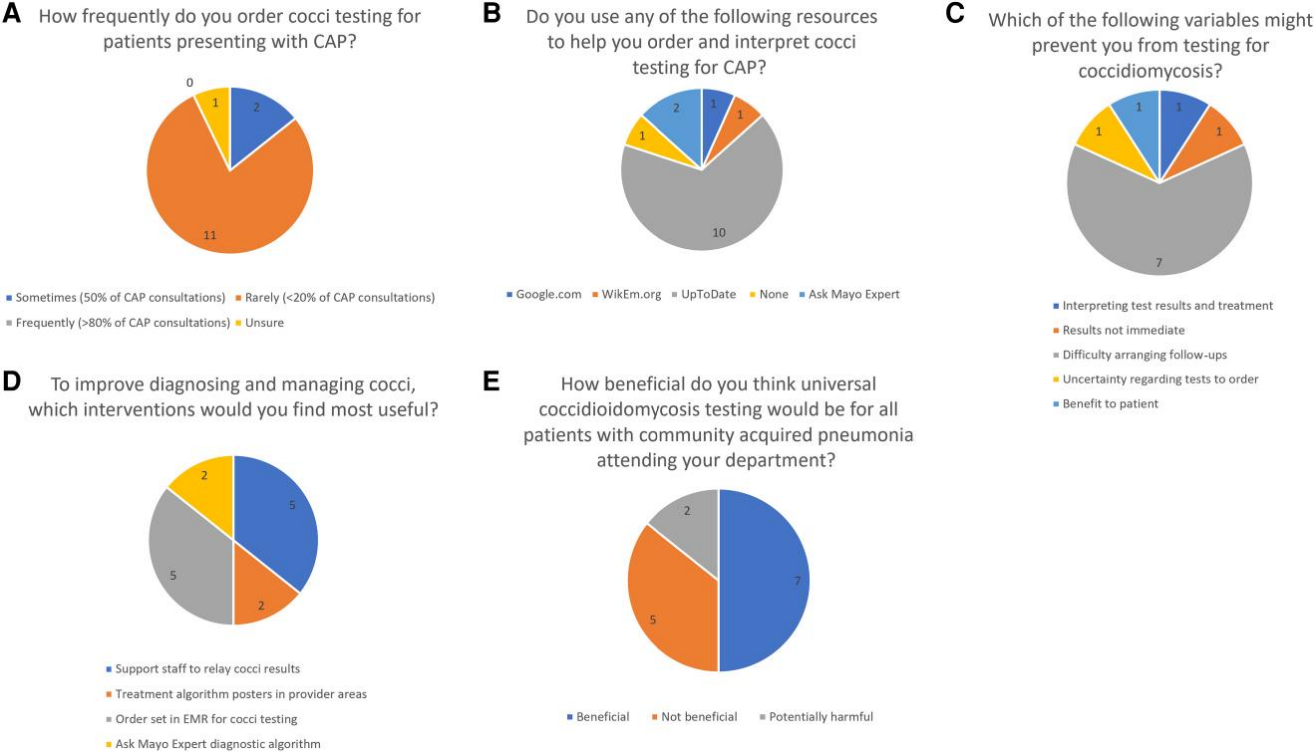
Survey of emergency department physicians on attitudes and practices concerning coccidioidal serologic testing in patients with suspected community-acquired pneumonia (CAP). Abbreviation: EMR, electronic medical record.

Of 37 ED physicians from our tertiary medical center who were sent the 12-item survey on attitudes toward CST, 14 (37.8%) responded. Most respondents (78.5%) rarely (in <20% of suspected CAP cases) ordered CST in patients with suspected CAP, with a minority ordering CST sometimes (in 50% of suspected CAP cases) or “unsure” regarding ordering frequency (Figure 1*A*). Most respondents (71.4%) used UpToDate.com for help with understanding which serologic tests to order and how to interpret the results (Figure 1*B*).

The main barrier to CST by ED physicians in our survey was difficulty arranging follow-up (50% of respondents; Figure 1*C*). Other barriers included results not being immediately available, uncertainty as to which serologic tests to order and how to interpret results, and the questionable benefit to the patient tested for coccidioidomycosis (each cited by 1 respondent). When asked which interventions could be implemented to improve CST rates in the ED, many respondents suggested support staff to relay coccidioidal results and an order set in the EMR for coccidioidal testing (each cited by 35.7%; Figure 1*D*). Other suggested interventions include a treatment algorithm posted in provisor areas (2 respondents) and an “Ask Mayo Expert” (https://askmayoexpert.mayoclinic.org/) diagnostic algorithm (2 respondents). When asked about the benefit of universal CST for patients with suspected CAP, 50% of respondents thought it would be beneficial, 35.7% thought it would be not beneficial, and 14.2% thought it would be potentially harmful (Figure 1*E*).

**Figure 2.**
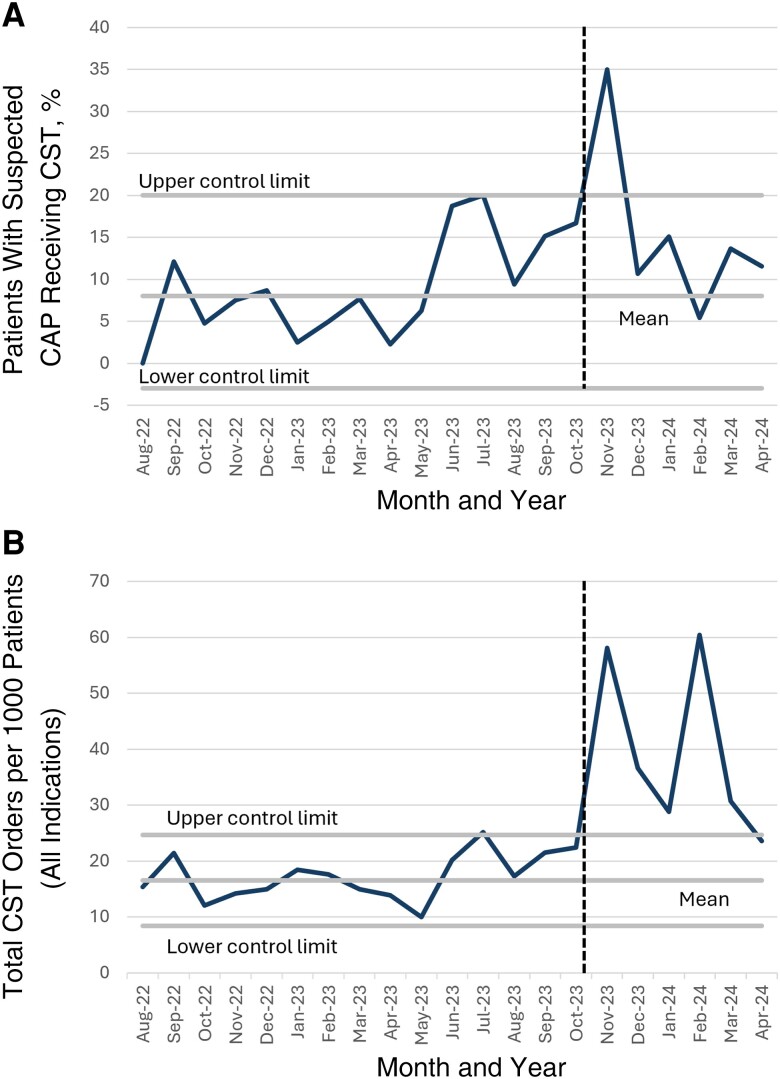
*A*. Percentage of patients with suspected community-acquired pneumonia (CAP) who received coccidioidal serologic testing (CST) before and after intervention (*vertical dashed line*). *B,* Number of CST orders (for any indication) per 1000 emergency department patients, before and after intervention (*vertical dashed line*). Abbreviations: 23, 2023; 24, 2024.

Next, we assessed the baseline CST rates in our tertiary ED. In line with previous studies showing low rates of CST in the ED setting, we found that CST was ordered in 8% (2 SDs, 0%–20%) of patients with suspected CAP (Figure 2*A*). This equated to an average of 16.5 CST orders per 1000 patients per month (2 SDs, 8.4–24.7; Figure 2*B*). We then deployed a targeted intervention aimed at improving the rates of CST within our ED, which included the CDC-sponsored “Community-Acquired Pneumonia: When To Think Fungus” module on 1 November 2023. After this intervention, we saw a significant (but nonsustained) improvement in the percentage of patients with suspected CAP who received CST (Figure 2*A*). We also saw a sustained increase in the number of CST orders for all indications (Figure 2*B*). These data suggest that targeted educational interventions are capable of increasing the CST rate in a tertiary medical center ED.

## DISCUSSION

In the current study, we found that ED physicians, even within a highly endemic region, had significant knowledge gaps regarding the pathophysiology of *Coccidioides* and reservations regarding CST. Most concerns were focused on the delay in obtaining CST results, uncertainty regarding the appropriate test(s) to order, and patient follow-up. Our survey results highlight potential interventions to improve the rate of CST. ED providers had significant concern regarding the delay between sample acquisition and result with current serologic testing. To address this concern, point-of-care assays have been developed; one was not sensitive enough to be useful [12], and another is undergoing testing in the clinical setting [13].

In addition, ED physicians had questions regarding which CST to order and the appropriate interpretation of the result. Several physicians favored the development of an order set or order panel, embedded into the EMR, that includes CST along with other tests commonly ordered for patients with suspected CAP (including chest radiography, complete blood cell count, and metabolic panel). Deployment of order sets and quick lists has been successful in increasing appropriate ordering for other common ED chief concerns, including exacerbation of congestive heart failure or chronic obstructive pulmonary disease and psychiatric concerns [14]. We are currently working on efforts to simplify CST nomenclature, update the ED order set to include CST, and add EMR best practice alerts to suggest CST if CAP is considered.

Finally, ED physicians had concerns regarding long-term patient follow-up. Within our institution, the establishment of a “coccidioidomycosis clinic,” staffed by an infectious disease physician, has been helpful for arranging follow-up in patients with coccidioidomycosis diagnosed in our ED. We have found that follow-up with a provider well versed in interpreting CST results is particularly important in light of the high rate of false-positive *Coccidioides* IgM results by enzyme immunoassay [6, 7]. Our team also created a list of potential outside providers who would be able to see patients in follow-up. Additional solutions are needed for underinsured or uninsured patients, who struggle to find outpatient follow-up.

In line with our survey results showing that the majority of ED physicians ordered CST infrequently (in <20% of suspected CAP cases), our preintervention period showed that CST was ordered for 8% of patients with suspected CAP. This rate increased transiently to 35% in the month following our targeted intervention but then returned to the preintervention baseline. We did see a sustained increase in the absolute number of CST orders for all indications (mean, 16.56 vs 19.9 per 1000 patients per month).

The current work does have several limitations. First, because only 37.8% of ED physicians responded to the survey, the survey may not capture the full spectrum of physician opinion regarding CST. This was a single-center study, so attitudes toward CST and the effectiveness of our educational intervention may not be generalizable. In addition, our data represent the percentage of patients with suspected CAP who received CST; therefore, other manifestations of coccidioidomycosis, such as erythema nodosum [4], were not specifically assessed. Finally. we did not measure the frequency of positive CST results in patients with suspected CAP, however, recent work suggests a higher rate in the summer months [15].

In conclusion, this work identified several barriers that prevent ED physicians in a highly endemic region from obtaining CST in patients with suspected CAP. It also identified several future interventions for improving the rate of testing. While a targeted educational intervention is capable of increasing CST orders by ED physicians, the gains are not sustained long term, arguing that systems-based approaches are needed.
